# Harnessing originally robust yeast for rapid lactic acid bioproduction without detoxification and neutralization

**DOI:** 10.1038/s41598-022-17737-4

**Published:** 2022-08-11

**Authors:** Radityo Pangestu, Prihardi Kahar, Lutfi Nia Kholida, Urip Perwitasari, Ahmad Thontowi, Fahrurrozi, Puspita Lisdiyanti, Yopi, Chiaki Ogino, Bambang Prasetya, Akihiko Kondo

**Affiliations:** 1 Graduate School of Engineering, Kobe University, 1-1 Rokkodaicho, Nada-ku, Kobe, 657-8501 Japan; 2 National Research and Innovation Agency (BRIN), Jl. Raya Bogor Km 46, Cibinong, Bogor, West Java 16911 Indonesia; 3 National Standardization Agency of Indonesia (BSN), Gedung Badan Pengkajian Dan Penerapan Teknologi (BPPT), Jl. M.H. Thamrin No. 8, Jakarta, 10340 Indonesia; 4 Graduate School of Science, Technology, and Innovation (STIN), Kobe University, 1-1 Rokkodaicho, Nada-ku, Kobe, 657-8501 Japan

**Keywords:** Industrial microbiology, Metabolic engineering, Biotechnology, Microbiology

## Abstract

Acidic and chemical inhibitor stresses undermine efficient lactic acid bioproduction from lignocellulosic feedstock. Requisite coping treatments, such as detoxification and neutralizing agent supplementation, can be eliminated if a strong microbial host is employed in the process. Here, we exploited an originally robust yeast, *Saccharomyces cerevisiae* BTCC3, as a production platform for lactic acid. This wild-type strain exhibited a rapid cell growth in the presence of various chemical inhibitors compared to laboratory and industrial strains, namely BY4741 and Ethanol-red. Pathway engineering was performed on the strain by introducing an exogenous *LDH* gene after disrupting the *PDC1* and *PDC5* genes. Facilitated by this engineered strain, high cell density cultivation could generate lactic acid with productivity at 4.80 and 3.68 g L^−1^ h^−1^ under semi-neutralized and non-neutralized conditions, respectively. Those values were relatively higher compared to other studies. Cultivation using real lignocellulosic hydrolysate was conducted to assess the performance of this engineered strain. Non-neutralized fermentation using non-detoxified hydrolysate from sugarcane bagasse as a medium could produce lactic acid at 1.69 g L^−1^ h^−1^, which was competitive to the results from other reports that still included detoxification and neutralization steps in their experiments. This strategy could make the overall lactic acid bioproduction process simpler, greener, and more cost-efficient.

## Introduction

Lactic acid is currently one of the most important chemical commodities due to widespread commercial applications in the pharmaceutical, cosmetics, chemical, and food industries^1^. With 400,000 tons of global production per year, lactic acid is considered a top-value platform chemical^2,3^. Moreover, lactic acid is the key precursor of poly-lactic acid—a popular biodegradable plastic with physicochemical, thermal, and mechanical properties comparable to typical petroleum-based polymers, such as polypropylene (PP) and low-density polypropylene (LDPE)^4,5^. Almost 90% of industrial lactic acid has been manufactured via fermentation rather than chemical synthesis^6^ as the former strategy is more environmentally friendly, less energy-intensive, and yields an optically pure product. Production of lactic acid is even more cost-effective when a low-cost feedstock is employed in the process. Due to its abundant availability, lignocellulosic biomass has been widely used as a substrate for producing various bio-based chemicals^7–9^. Besides, sugarcane bagasse (SCB) generated by the sugar and alcohol industry is ideal for this objective. Data show that more than 1.8 billion tons of sugarcane were produced around the world in 2017^10^, with bagasse accounting for 31.8% of the sugarcane composition^11^. Considering its availability, the utility of SCB has become the subject of numerous studies related to establishing a circular bio-economy and sustainable industries.

Despite the compelling benefits, utilizing lignocellulose as a feedstock for bioprocessing has several bottlenecks. The generation of various by-products during the pretreatment process is one of the challenging issues. These by-products, which include furan derivatives (furfural, 5-HMF, etc.), weak organic acids (formic acid, acetic acid, etc.), and phenolic compounds (vanillin, ferulic acid, etc.), inhibit microbial metabolism^12^, which renders fermentation and diminishes productivity. Biological, physical, and chemical methods have been explored in a quest to detoxify these chemicals^13^. However, those methods necessitate additional equipment, which drives up cost, and reduce the quantity of fermentable sugar in the hydrolysate^14^. Therefore, employing a stress-tolerant microorganism in the fermentation step would undoubtedly be more desirable than performing additional detoxification steps.

During lactic acid bioproduction, acidic products, including the target product itself, may cause significant stress for a microbial host. Many microorganisms, particularly bacteria, suffer growth-rate inhibition under highly acidic conditions. Commonly, a neutralizer, such as calcium carbonate, is added to maintain the pH of the medium. However, in addition to increasing cost, some of these neutralizing agents are toxic for microorganisms and react with the fermentation products to form insoluble calcium salts that can easily mix with biomass and complicate the subsequent downstream process. This process generates the target product as calcium lactate instead of its (free) acid form. Consequently, a recovery step by acidification, which increases the total operating cost of lignocellulosic lactic acid production^15^, is needed to obtain lactic acid. Most importantly, this acidification process generates a by-product, gypsum, in a large quantity (1 ton per ton of lactic acid production^6^), which must be disposed of in landfills^16^ and, therefore, magnifies the environmental burden. Based on a life-cycle assessment (LCA), operational systems comprising the neutralization-acidification steps exhibited higher environmental impacts related to climate change, freshwater eutrophication, terrestrial acidification, etc., compared to process scenarios catalyzed by an acid-tolerant microorganism^15^. Hence, from both economic and ecological perspectives, eliminating neutralizing agents by applying a strong microbial host would be advantageous.

Various approaches to obtaining a robust microorganism have been proposed. Tolerance engineering by genetic modification is an example of common tools to enhance strain robustness. For instance, the co-expression of *TAL1* and *ADH1* in *Saccharomyces cerevisiae* enhances ethanol production in a medium containing furfural^17^. Co-overexpression of *HAA1* and *PRS3* or disruption of *FPS1* could also improve acetic acid tolerance^18,19^. Nevertheless, due to the complexity of biomass chemical composition, a large number of tolerance-related genes must be simultaneously introduced to the microorganism of interest^20–22^, making this approach cumbersome.

On the contrary, the strategy proposed here focuses on increasing the lactic acid production of a naturally robust microorganism engineered rather than performing extensive genetic engineering. In the present study, we selected newly isolated yeast, identified as *S. cerevisiae* BTCC3, obtained from screening Ascomycota yeasts deposited in the Indonesian Culture Collection (InaCC). This strain can survive at low pH and in the presence of lignocellulose-derived chemical inhibitors, such as furfural, formic acid, acetic acid, and other inhibitors. However, similar to other yeasts, this strain lacks the metabolic pathway for lactic acid generation. Therefore, we introduced an exogenous L-*LDH* gene to enable the lactic acid fermentation from glucose as an example. This experiment intended to construct a microbial strain with phenotypes suitable for utilizing lignocellulosic biomass as a low-cost carbon feedstock, such as SCB, with high tolerance to acidic and chemical inhibitor stresses. Also, we considered the potential of this recombinant strain to ferment glucose to lactic acid without detoxifying and neutralizing treatments.

## Results

### Strain performance in tolerating chemical inhibitors

**Figure 1:**
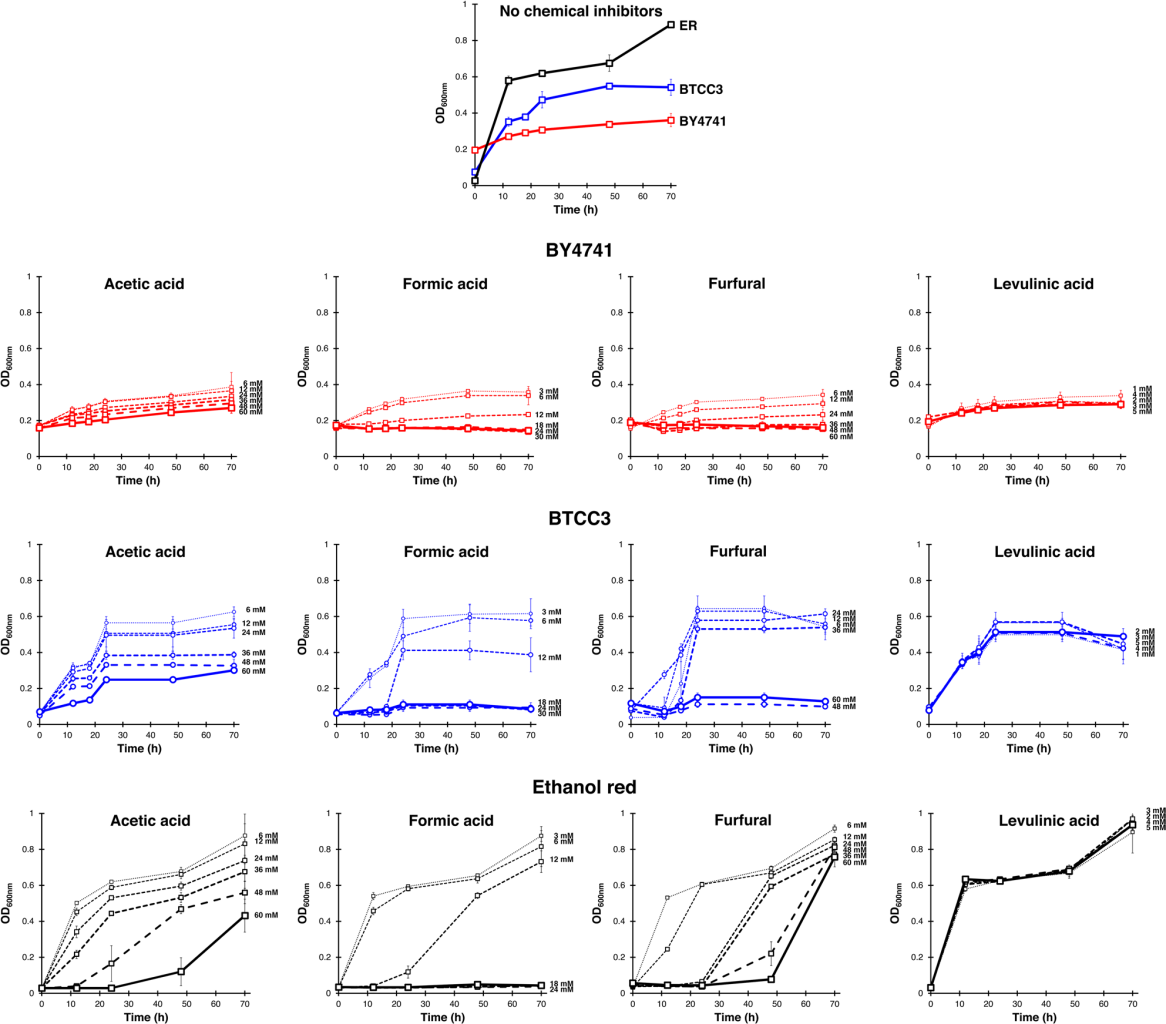
The cell growth of *Saccharomyces cerevisiae* BTCC3 (blue boxes), BY4741 (red boxes) and Ethanol-red (black boxes) strains in YNB medium + amino acids with and without single chemical inhibitors supplemented. Values represent the average measurement of three biological replicates. Error bars represent the standard deviation of measurements.

Firstly, we compared the cell growth of our robust strain, *S. cerevisiae* BTCC3, with a common laboratory strain of *S. cerevisiae*, BY4741, and an industrial strain possessing high robustness, Ethanol-red. We initially performed the cultivation using a minimum synthetic medium without amino acid. Although BTCC3 strain could grow well in this medium, BY4741 strain exhibited no significant growth even when no chemical inhibitor was added. Therefore, we used amino-acids-supplemented medium instead to compare the growth rates of the three yeast strains. As shown in Fig. 1, the BTCC3 strain displayed a considerably higher cell growth compared to the BY4741 strain in the medium with no chemical inhibitor. Since acetic acid, formic acid, furfural and levulinic acid are the most common by-products generated during the pretreatment of various lignocellulosic biomass, these chemicals were added to the medium of fermentation to assess the robustness of both strains. The effect of each chemical inhibitor on cell growth was studied at various levels, viz., 0%, 10%, 20%, 40%, 60%, 80% and 100%, in corresponding with the original concentrations of inhibitory chemical complexes (ICC) examined elsewhere^23,24^. BTCC3 strain remarkably grew faster under all four chemicals, including acetic acid, formic acid, furfural and levulinic acid, compared to BY4741 strains. However, cell growth inhibition in BTCC3 was noticeable when formic acid and furfural reached the concentrations of 18 mM and 48 mM, respectively. At those concentrations, the OD_600nm_ appeared to drop around 80% relative to the values for cultivation with no inhibitor. Despite growing faster than BTCC3 in the absence of chemical inhibitor, Ethanol-red strain displayed noticeable decreases when acetic acid, formic acid, and furfural reached the concentrations of 60 mM, 18 mM, and 24 mM, respectively, as the values of OD_600nm_ dropped up to 80% at those levels relative to the cultivation with no chemical inhibitors. Therefore, in comparison to Ethanol-red strain, BTCC3 might possess slightly higher tolerance limits towards acetic acid and furfural.

**Figure 2:**
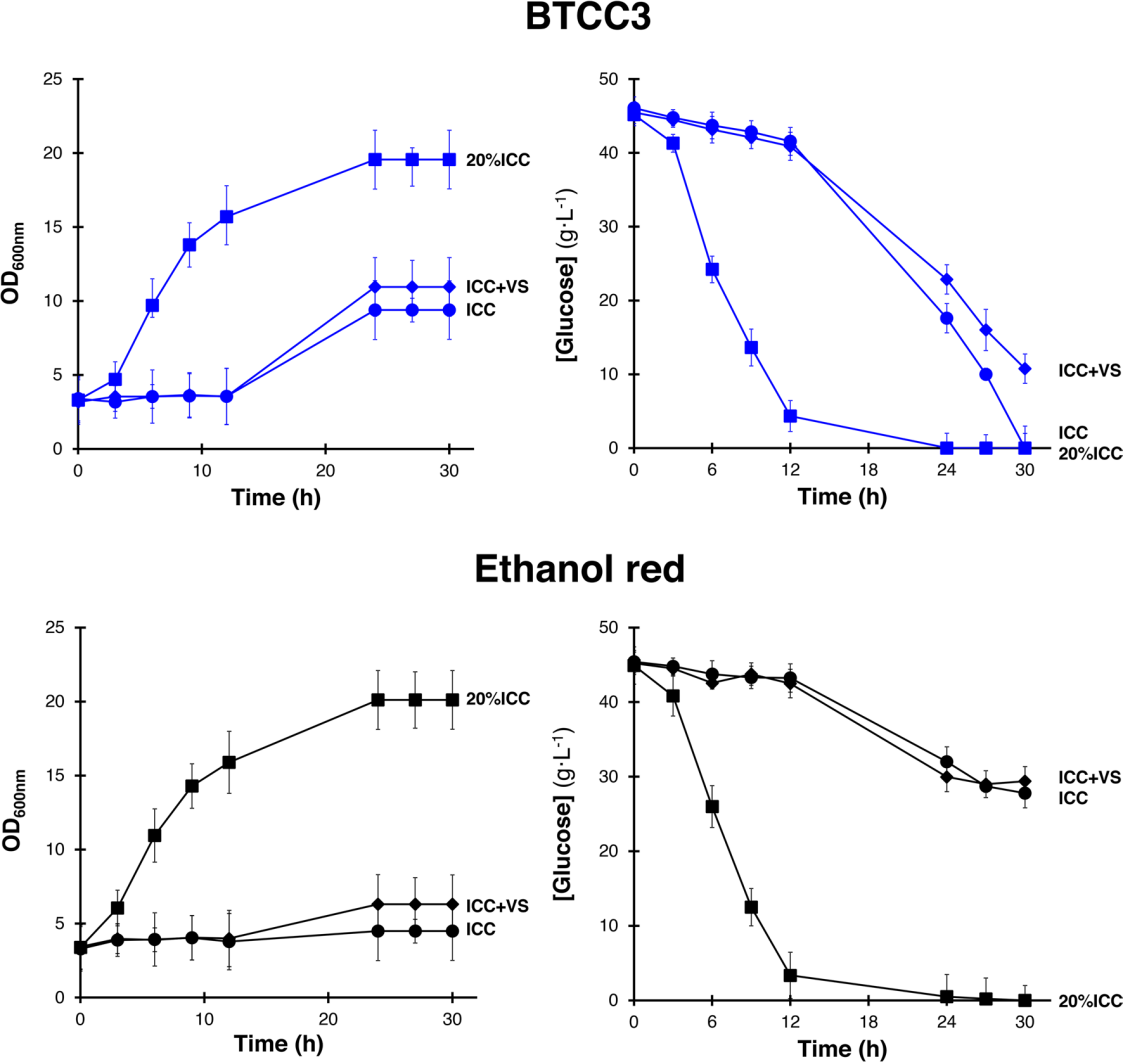
The cell growth and rate of glucose uptake of *Saccharomyces cerevisiae* BTCC3 strain (blue lines) and Ethanol-red (black lines) in YNB medium supplemented with ICC at 20 % level (20%ICC; squares), ICC at 100% level (ICC; circles), and ICC at 100% level with the addition of 0.6 mM vanillin and 0.4 mM syringaldehyde (ICC +  VS; diamonds). Values represent the average measurement of three biological replicates. Error bars represent the standard deviation of measurements.

Subsequently, we also performed fermentation using medium supplemented with ICC^23﻿^ (consisting of acetic acid, formic acid, 5-HMF and levulinic acid) at levels of 20% and 100% to analyze whether BTCC3 and Ethanol-red could tolerate chemical inhibitors in a combination form. We also evaluated the robustness of both strains against vanillin and syringaldehyde (VS), two common chemicals formed from lignin degradation, by fermenting both using medium supplemented with ICC plus 0.6 mM vanillin and 0.4 mM syringaldehyde (ICC + VS). With a higher initial cell concentration, both strains could still grow in YNB medium with 2 0% level of ICC (Fig. 2). However, Ethanol-red strain grew slower than BTCC3 strain when ICC at 100% level and VS were supplemented into the medium. In addition, the decelerating effects of VS addition and an increased ICC level on the rate of glucose uptake were also more apparent in Ethanol-red strain within 30 h of cultivation compared to BTCC3 strain. According to these results, we assumed that BTCC3 could be a good candidate for robust industrial strain that can tolerate a mixture of chemical inhibitors contained in various lignocellulosic hydrolysates without requiring to conduct much detoxification treatment prior to fermentation.

### Pathway manipulation and its effect on the yeast metabolism

**Figure 3:**
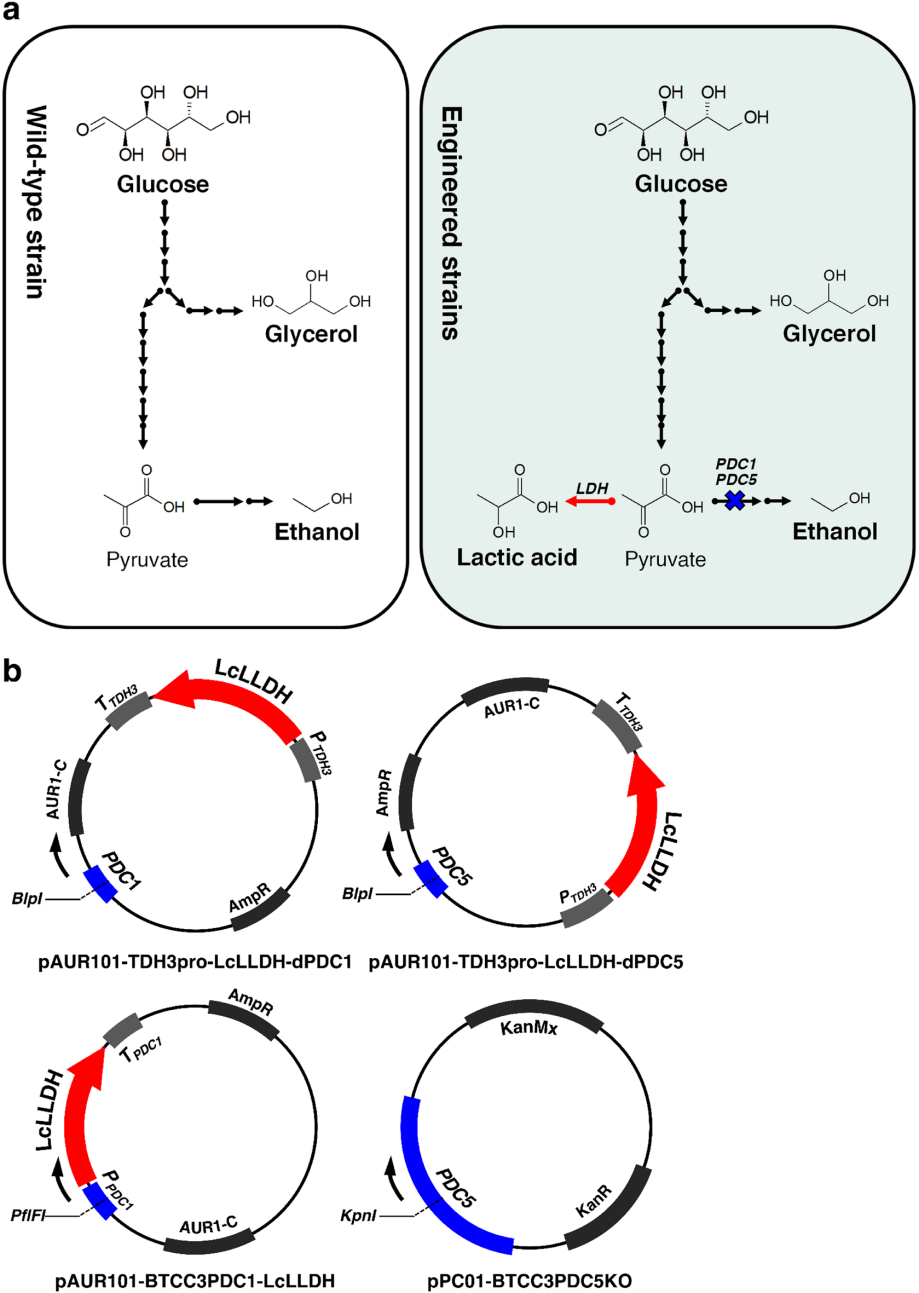
Pathway engineering in *Saccharomyces cerevisiae* BTCC3 by introducing an exogenous *LDH* gene to enable lactic acid generation and disrupting *PDC1/5* genes to reduce metabolic flux to ethanol (**a**) using genome-integrative plasmids shown (**b**). *BlpI*, *PflFI* and *KpnI* indicate restriction enzymes used to digest the plasmids. Blue regions indicate the overlapping sequence of *PDC 1/5* genes to the genome.

As previously described, the BTCC3 (wild-type) strain did not produce lactic acid due to the lack of a metabolic pathway for lactic acid production from sugar. Here, we inserted an exogenous *LDH* gene and disrupted several *PDC* genes (Fig. 3a). Since introducing the *LDH* gene into *S. cerevisiae* genome using a genome-integrated plasmid yields a higher accumulation of lactic acid than using an episomal-type plasmid^25^, the former method was used to construct all engineered strains in this experiment. For the simultaneous expression of the *LDH* gene and disruption of *PDC* genes, partial coding sequences of *PDC1* and *PDC5* genes (later shown as *PDC1/*5) were cloned into plasmids containing an *LDH* expression cassette (Fig. 3b). The sequence of *LDH* was fused with the *TDH3* promoter and terminator sequences at both its upstream and downstream regions, respectively, to construct constitutively expressed *LDH* systems, namely pAUR101-TDH3pro-LcLLDH-dPDC1 and pAUR101-TDH3pro-LcLLDH-dPDC5. There could be a possibility that *TDH3p* and *PDC1/5p* simultaneously control the expression of the *LDH* gene after integrating gene-expressing cassettes into the genome. Hence, the orientation of *TDH3p*-*LcLLDH*-*TDH3t* was flipped in both systems to avoid the contribution of the *PDC1/5* promoter to the expression of *LDH* gene. Moreover, pAUR101-BTCC3PDC1-LcLLDH has a sequence of *LDH* gene integrated between the promoter and terminator of *PDC1* gene cloned from the genome to intentionally utilize this native glucose-dependent promoter. In contrast, pP01-BTCC3PDC5KO contains only the partial coding sequence of *PDC*5 and a marker gene to distort the expression of the *PDC5* without introducing the *LDH* gene. Transformation of these plasmids into BTCC3 yielded five BTCC3-derived yeasts, including LX1, LX5, LA1, LA15 and LA2 strains (Table 1).

**Table 1:** List of strains and plasmids used in this study.

Strain/plasmid	Genotype and description	Source
***S. cerevisiae***		
BTCC3	Wild type, haploid	InaCC
BTCC3 LX1	∆*PDC1*::*P*_*TDH3*_–*LcLLDH*–*T*_*TDH3*_ BTCC3 harboring pAUR101-TDH3pro-LcLLDH-dPDC1	This study
BTCC3 LX5	∆*PDC5*:: *P*_*TDH3*_–*LcLLDH*–*T*_*TDH3*_ BTCC3 harboring pAUR101-TDH3pro-LcLLDH-dPDC5	This study
BTCC3 LA1	∆*PDC1*::*P*_*TDH3*_–*LcLLDH*–T_*TDH3*_ ∆*PDC5* BTCC3 harboring pAUR101-TDH3pro-LcLLDH-dPDC1 and pPC01-BTCC3PDC5KO	This study
BTCC3 LA15	∆*PDC1*::*P*_*TDH3*_–*LcLLDH*–*T*_*TDH3*_ ∆*PDC5*::*P*_*TDH3*_–*LcLLDH*–*T*_*TDH3*_ BTCC3 harboring pAUR101-TDH3pro-LcLLDH-dPDC1 and pAUR101-TDH3pro-LcLLDH-dPDC5	This study
BTCC3 LA2	∆*PDC1*::*LcLLDH* ∆*PDC5* BTCC3 harboring pAUR101-BTCC3PDC1-LcLLDH and pPC01-BTCC3PDC5KO	This study
**Plasmids**		
pAUR101-BTCC3PDC1-LcLLDH	*P*_*PDC1*_–*LcLLDH*–*T*_*PDC1*_, *AMP*^*R*^, *AUR1-C*	This study
pAUR101-TDH3pro-LcLLDH-dPDC1	*PDC1*, *P*_*TDH3*_–*LcLLDH*–T_*TDH3*_, *AMP*^*R*^, *AUR1-C*	This study
pPC01-BTCC3PDC5KO	*PDC5*, *KAN*^*R*^, *KanMx*	This study
pAUR101-TDH3pro-LcLLDH-dPDC5	*PDC5*, *P*_*TDH3*_–*LcLLDH*–*T*_*TDH3*_, *AMP*^*R*^, *AUR1-C*	This study

**Figure 4:**
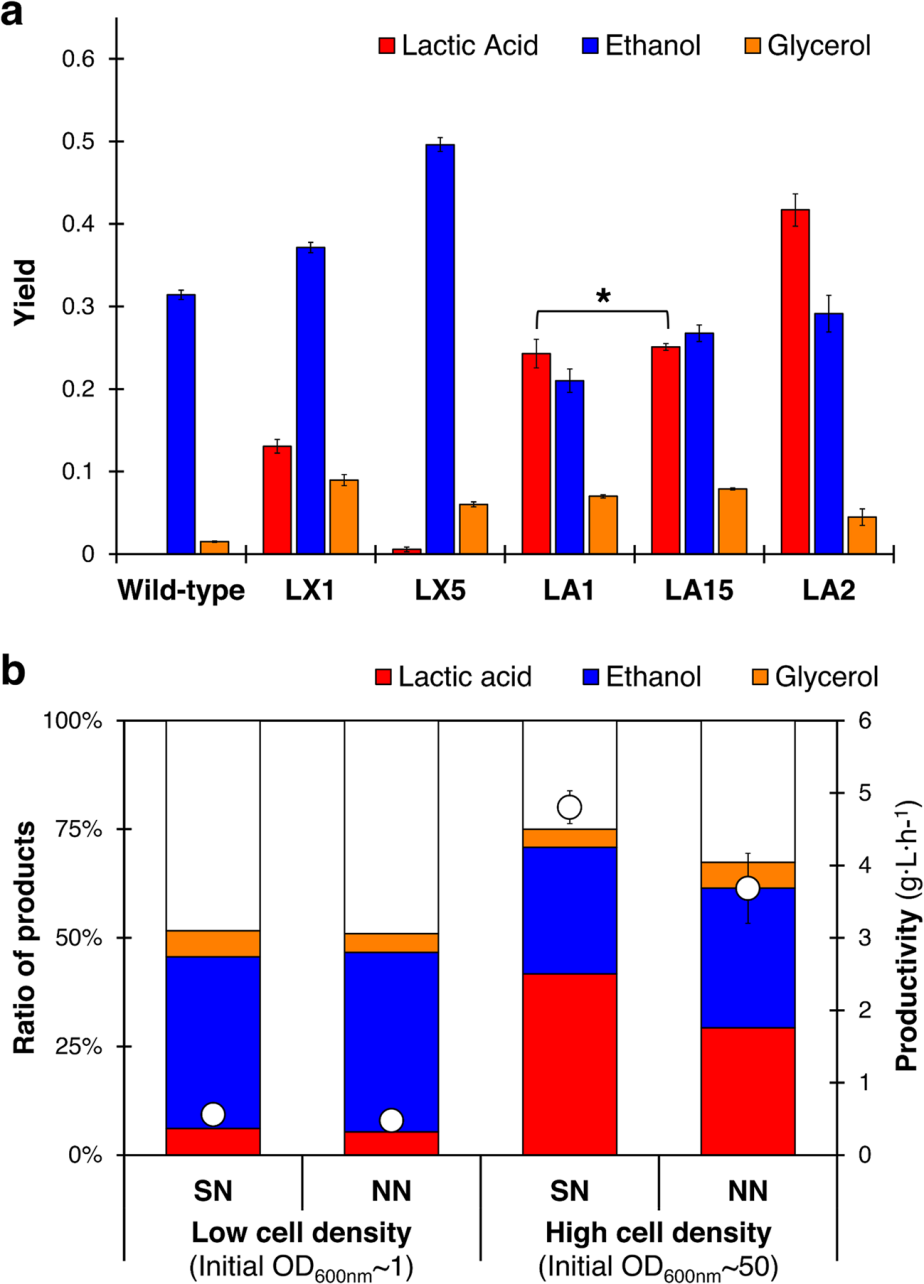
The effect of pathway engineering on the fermentation products of *Saccharomyces cerevisiae* BTCC3 (**a**) and cell density on ratio of products and lactic acid productivity in *Saccharomyces cerevisiae* BTCC3LA2 under semi-neutralized (SN) and non-neutralized (NN) cultivations (**b**). Values represent the average measurement of four biological replicates. Error bars represent the standard deviation of measurements. Bars indicated by an asterisk represent a statistically insignificant correlation (Student’s t-test, *P* ≤ 0.05).

Figure 4a shows how this pathway adjustment affected the product accumulation. As expected, the insertion of *LDH* genes enhanced the production of lactic acid in all mutant strains. In semi-neutralized (SN) high cell density cultivation, LX1 strain (*PDC1*^−^, *LDH*^+^) generated a 20-fold higher level of lactic acid compared with the results using LX5 strain (*PDC5*^−^, *LDH*^+^). Our results also revealed that the generation of lactic acid elevated the accumulation of ethanol and glycerol because both mutant strains exhibited higher levels of production compared with the levels from wild-type strains. Moreover, two PDC genes were knocked out to generate the LA1 strain (*PDC1*^−^, *PDC5*^−^, *LDH*^+^). This strain exhibited a 1.9-fold increase in lactic acid generation and a 1.8-fold decrease in ethanol accumulation compared with LX1 strain (*PDC1*^−^, *LDH*^+^). Surprisingly, the concentration of lactic acid produced from two copies of the LDH gene-harboring strain, LA15 strain (*PDC1*^−^, *LDH*^+^, *PDC5*^−^, *LDH*^+^), showed no significant difference (Student’s t-test, *P* ≤ 0.05) compared to LA1 strain (*PDC1*^−^, *LDH*^+^, *PDC5*^−^). We also conducted further genetic modification to disrupt other ethanol-related genes, namely *PDC6* and *ADH1*, as a way of completely blocking the conversion of pyruvate to ethanol. However, no colonies were obtained after transformation. This result agreed with other studies that found disrupting those genes results in severe cell growth impairment. Triple deletion of *PDC1*, *PDC5* and *PDC6* genes inhibited cell growth because these genes are essential for NAD^+^ production^26^. A study also show that the *PDC6* gene shows the lowest expression among the three, and mere deletions of *PDC1* and *PDC5* would be sufficient to significantly diminish the specific activity of pyruvate decarboxylase^27^. Meanwhile, the deletion of *ADH1* is known to slow the rate of cell growth due to the accumulation of toxic acetaldehyde^28^.

The effect of promoter strength on the production of lactic acid in this strain was also examined. In addition to the LA1 strain (*PDC1*^−^, *PDC5*^−^, *TDH3p*-*LDH*^+^) that possesses *LDH* gene integrated to a constitutive promoter, LA2 strain (*PDC1*^−^, *PDC5*^−^, *PDC1p*-*LDH*^+^) containing an *LDH* gene with glucose-dependent promoter was also constructed. Interestingly, our result revealed that the LA2 strain produced 43.23 g L^−1^ of lactic acid (approximately two times higher than the LA1 strain). This result indicated that the use of a constitutive promoter to express the *LDH* gene might not be suitable for our strain, although this type of promoter is commonly used in numerous experiments due to its high level of expression^29–31^. Meanwhile, the LA2 strain harboring the *LDH* gene under the control of a glucose-dependent promoter showed the highest lactic acid production. There are several plausible rationalizations for this result. It could simply be because *PDC1p* is a native promoter. In addition, although *TDH3p* is categorized as a constitutive promoter, its expression declines in the presence of ethanol^32^—one of the major products generated by all strains in this type of experiment. Also, it is worth noting that the employment of constitutive promoters could increase the metabolic load^33^, affecting the cell growth rate, metabolic flux, and redox balance of microbial hosts.

### Strain performance without a neutralizing agent

Adding a neutralizer into the medium at the beginning of fermentation, termed semi-neutralized (SN) cultivation, makes the overall process inefficient. Therefore, our engineered strain was intended to facilitate a completely non-neutralized (NN) cultivation setting. Since the LA2 strain could accumulate lactic acid at the highest concentration compared to other BTCC3-derived hosts, this strain was selected for further observation. Firstly, we evaluated the ability of this strain to generate lactic acid without a neutralizer in low cell density cultivation. As shown in Fig. 4b, this strain could still produce lactic acid without neutralizing treatment with only a 15% drop in productivity compared to the SN condition. However, in this low cell density cultivation, our strain utilized most of carbon source for cell growth and ethanol accumulation. Meanwhile, the accumulations of lactic acid were below 10 g L^−1^ under SN and NN conditions. Moreover, homofermentative lactic acid requires anaerobic condition. Our strategy attempted to limit the oxygen dissolved by increasing the initial cell concentration and setting the agitation at a low rate. Increasing the initial optical density (OD_600nm_) from 1 to 50 could boost lactic acid generation 5.7-fold higher than cultivation with lower initial cell concentration.

**Figure 5:**
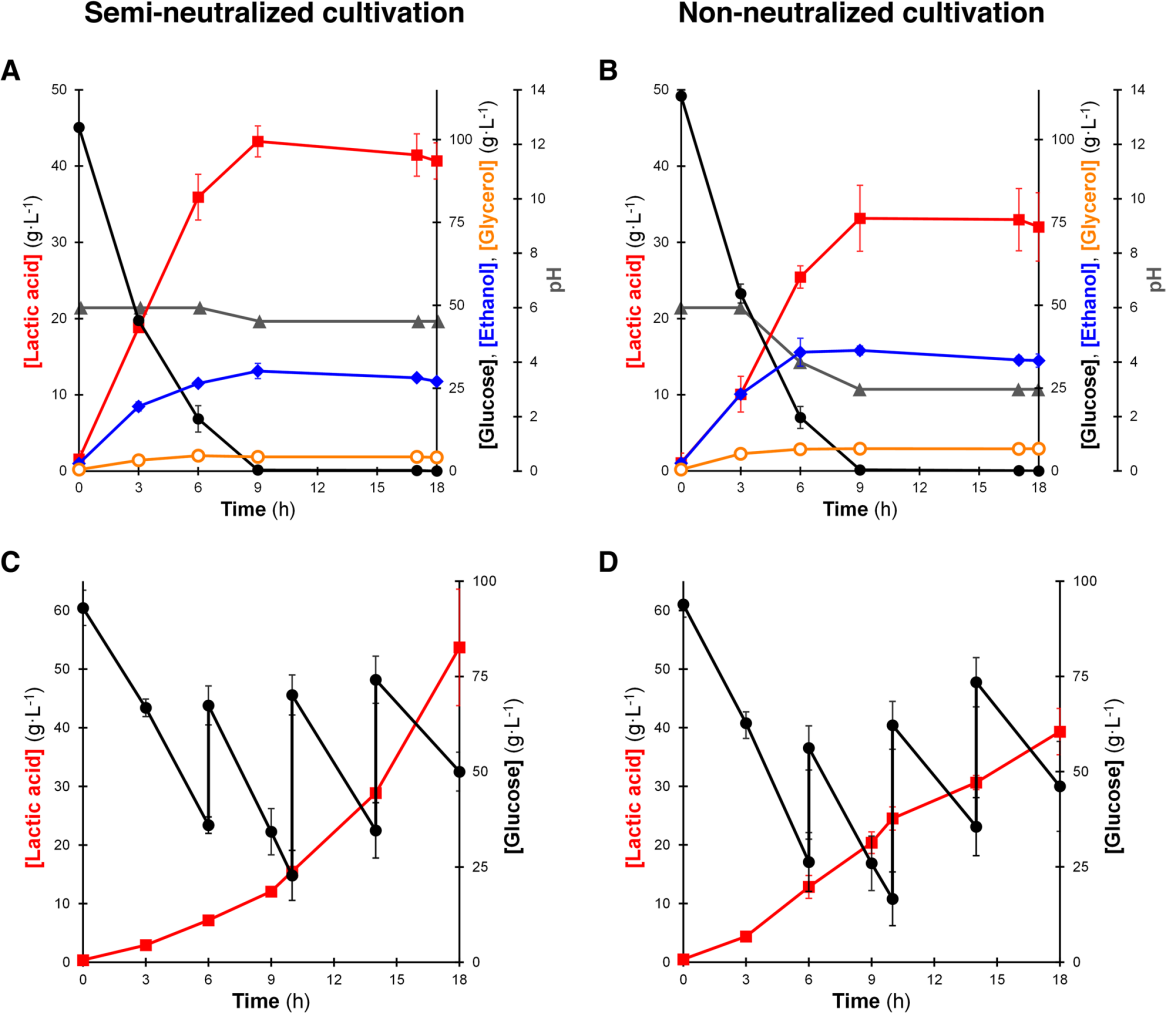
Fermentation products of *Saccharomyces cerevisiae* BTCC3LA2 strain using YPD medium under semi-neutralized (**A**,**C**) and non-neutralized (**B**,**D**) conditions in a batch (**A**,**B**) and fed batch (**C**,**D**) cultivation with the initial OD_600nm_ = 50. Line markers represent lactic acid (red boxes), glucose (black circles), ethanol (blue diamonds), glycerol (orange circles) and pH (grey triangles). Values represent the average measurement of four biological replicates. Error bars represent the standard deviation of measurements.

Figure 5A,B shows that with and without the addition of calcium carbonate (as a neutralizing agent), the LA2 strain produced lactic acid at 43.2 and 33.2 g L^−1^, respectively. Even though the accumulation of lactic acid declined under NN condition, the removal of calcium carbonate did not cause a significant decrease in the rate of glucose uptake as the glucose was completely consumed within only 9 h under both SN and NN conditions. Nonetheless, as shown in Table 2, these results were relatively competitive if compared with the productivity of other microbial hosts as previously reported^25,34–37^. Despite its lower titer and yield, the LA2 strain maintained the ability to utilize glucose rapidly and exhibited a lower drop in productivity under NN condition compared to other studies. With the addition of a neutralizing agent (SN), the productivity was 4.80 g L^−1^ h^−1^. Meanwhile, under NN condition, the productivity remained as high as 3.68 g L^−1^ h^−1^, despite experiencing a pH drop to 3.00 after 9 h of cultivation (∆pH =  ± 1.5, compared to SN cultivation). Mostly, other studies shown in Table 2 exhibited a drop in productivity by around 66% without supplementing neutralizing agent into the medium.

**Table 2:** Comparison of lactic acid fermentation with and without a neutralizing agent from several studies.

Microbial host	Strategies	Condition^a^	Time (h)	Titer (g L^−1^)	Yield (g g^−1^ glucose)	Productivity (g L^−1^ h^−1^)
*Pichia kudriavzevii* DKA^34^	Deletion of PDC1 and insertion of two copies of *LDH* Enhanced acid-tolerance by adaptive laboratory evolution Fed-batch strategy	SN (Ca(OH)_2_ to pH 4.7)	33	154	0.72	4.16
		NN	42	60.1	0.78	1.43 (− 66%)^b^
*Candida sonorensis* ATCC32109-derived mutant^35^	Deletion of *PDC1* and *PDC2,* and insertion of two copies of *PGK1p*-*LDH* from *L. helveticus*	SN (30 g L^−1^ CaCO_3_)	144	66	0.66	0.45
		NN	100	14	0.28	0.14 (− 66%)^b^
*S. cerevisiae* YIBO-7A^25^	Deletion of *PDC1* and insertion of two copies of *LDH* from Bovine	SN (50 g L^−1^ CaCO_3_)	42	55.6	0.62	1.11
		NN	72	50.6	0.51	0.70 (− 37%)^b^
*S. cerevisiae* δpHδLA2-51/dP36^36^	Deletion of *PDC1* and insertion of *LDH* from *Leuconostoc mesenteroides*	SN (20 g L^−1^ CaCO_3_)	23	52.2	0.55	2.27
		NN	52	33.9	0.29	0.65 (− 71%)^b^
*S. cerevisiae* BK01^37^	Insertion of *LDH* from *L. acidophilus* and introduction of xylose oxidoreductase pathway derived from *Pichia stipites* Enhanced lactic acid tolerance by adaptive laboratory evolution	NN	96	119	0.60	1.24
*S. cerevisiae* BTCC3 LA2 (this study)	Utilization of an originally robust strain Deletion of *PDC1* and *PDC5*, and insertion of only one copy of *PDC1p-LDH* from *Lactobacillus casei* High cell density cultivation	﻿Batch:				
		SN (50 g L^−1^ CaCO_3_)	9	43.2	0.43	4.80
		NN	9	33.2	0.33	3.68 (− 23%)^b^
		Fed-batch:				
		SN (50 g L^−1^ CaCO_3_)	18	53.7	0.33	2.98
		NN	18	39.3	0.24	2.18 (− 27%)^b^

^a^*SN* semi-neutralized, *NN* non-neutralized.

^b^The percentage values indicate the drop in productivity when no calcium carbonate was added at the beginning of fermentation.

Based on the results shown in Fig. 5A,B, lactic acid production was terminated because the glucose was completely consumed within 9 h. However, as depicted in Fig. 5C,D, the LA2 strain could still accumulate lactic acid when additional glucose was supplemented into the medium even without neutralizing treatment. After being fed three times with concentrated glucose at 6 h, 10 h and 14 h, the productivity of engineered strain stood at 2.98 g L^−1^ h^−1^ ([LA]_18 h_ = 53.7 g L^−1^) and 2.18 g L^−1^ h^−1^ ([LA]_18 h_ = 39.3 g L^−1^) under SN and NN conditions, respectively. Similar to the results from batch cultivation, this strain exhibited stable productivity under both conditions, with a drop in value less than 30% under NN cultivation.

### Fermentation using bagasse hydrolysate

Previously, BTCC3 showcased a strong tolerance to several chemical inhibitors both in an individual and combination form compared to other two strains. However, assessing its performance using a real lignocellulosic hydrolysate is also essential. Therefore, the hydrolysate obtained from sugarcane bagasse (SCB) pretreatment via hot water method was directly used as a medium without prior treatment of enzymatic hydrolysis, detoxification, or neutralization. This medium contained 50 g L^−1^ glucose, 25 g L^−1^ xylose, furfural (4.5 mM), 5-HMF (3.4 mM), acetic acid (66 mM), formic acid (7.2 mM), and lactic acid (7 mM) (initial pH = 4.5). Although xylose was also present in the hydrolysate, our yeast strain possesses no active genes that connect the lactic acid generation and xylose metabolism pathways. Therefore, the concentrations of xylose were not included in any of the calculations.

**Figure 6:**
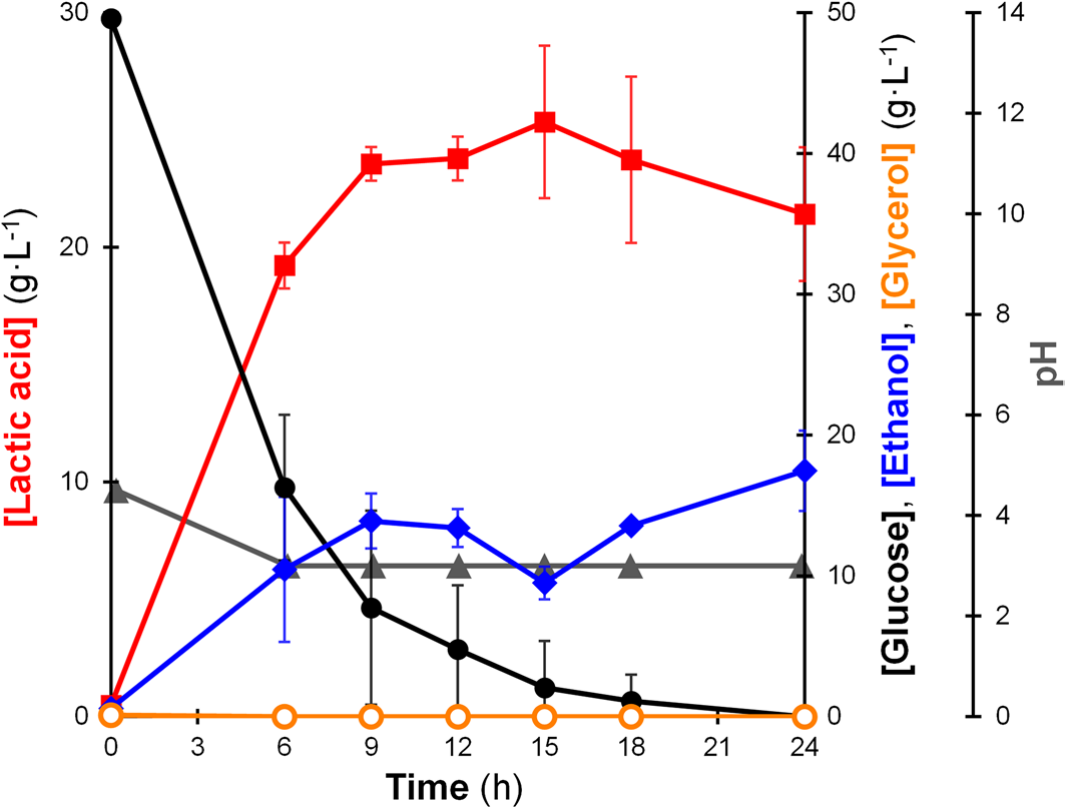
Fermentation products of *Saccharomyces cerevisiae* BTCC3LA2 strain using non-detoxified hydrolysate from sugarcane bagasse as the medium under non-neutralized condition with the initial OD_600nm_ = 50. Line markers represent lactic acid (red boxes), glucose (black circles), ethanol (blue diamonds), glycerol (orange circles) and pH (grey triangles). Values represent the average measurement of four biological replicates. Error bars represent the standard deviation of measurement.

As can be seen in Fig. 6, the LA2 strain generated lactic acid in a concentration of 25.34 ± 3.25 g L^−1^ at 15 h from an initial glucose concentration of 49.57 ± 0.49 g L^−1^ (yield = 0.51 g g^−1^ glucose; productivity = 1.69 g L^−1^ h^−1^). Despite lower titer and yield, the productivity of our strain was relatively close to the results from other studies attempting to valorize SCB using common lactic-acid-producing strains, such as *Bacillus coagulans* and *Lactobacillus pentosus*^38–41^ (Table 3). Besides, in those previous reports, sodium hydroxide still had to be added to maintain the pH of the medium during fermentation. Meanwhile, in our experiment, that level of productivity was achieved without the neutralizing treatment at any stage of fermentation. Interestingly, although cultivation using the SCB hydrolysate displayed declines in the rate of glucose uptake and productivity, the LA2 strain could convert about 51% of the initial glucose (Fig. 6), whereas its cultivation using the YPD medium converted only 33% of the glucose (Fig. 5A). Also, the ratio of lactic acid to ethanol was remarkably improved (2.67 at 15 h) compared with cultivation using a YPD medium (0.91 at 9 h). This result indicates that the stress of chemical inhibitors at up to certain levels could instead provide a positive impact to the fermentation by shifting the metabolism and restricting the cell growth^24^, which resulted in an increased lactic acid accumulation.

**Table 3:** Comparison of lactic acid fermentation using sugarcane bagasse as raw material from several studies.

Microbial host	Strategies	Time (h)	Titer (g L^−1^)	Yield (g g^−1^ glucose)	Productivity (g L^−1^ h^−1^)
*Bacillus coagulans* DSM2314^38^ (wild-type, bacteria)	Pretreated using acid and steam explosion method Cell adaptation using furfural prior to fermentation Enzyme hydrolysis using Genencor GC220 Fermentation at 50 °C, 100 rpm (1-L reactor) Two-stage simultaneous saccharification and fermentation pH control at 5 using Ca(OH)_2_ at the beginning and during fermentation	37	58.2	0.90	1.81
*Lactobacillus pentosus*^39^ (wild-type, bacteria)	Pretreated using acid and steam explosion method Cell adaptation using hydrolysate prior to fermentation Enzyme hydrolysis using Cellic C-TEC2 Fermentation at 37 °C, 200 rpm (1-L bioreactor) Fed-batch simultaneous saccharification and fermentation pH control at 6–6.5 using NaOH at the beginning and during fermentation	72	72.8	0.61	1.01
*B. coagulans* DSM ID 14-300^40^ (wild-type, bacteria)	Pretreated using acid method Bio-detoxification treatment Fermentation at 52 °C, 200 rpm (500-mL reactor) pH control at 6.0 using NaOH at the beginning and during fermentation	50	56.0	0.87	1.70
*B. coagulans* NCIM 5648^41^ (wild-type, bacteria)	Pretreated using alkali method and followed by washing process Enzyme hydrolysis using Cellic C-TEC2 Fermentation at 50 °C, 100 rpm pH control at 7.0 using NaOH during fermentation	24	69.2	0.76	2.88
*S. cerevisiae* BTCC3 LA2 (recombinant, yeast)	Pretreated using hot water No prior detoxification or enzyme hydrolysis processes Fermentation at 30 °C, 90 rpm, high-cell density cultivation (100 mL-flask) No supplementation of a neutralizing agent at any stage of fermentation	15	25.34	0.51	1.69

## Discussion

Establishing a strong microbial host can simplify the overall process of lactic acid bioproduction because additional treatments, such as detoxification and neutralization, can be eliminated. Rather than employing extensive tolerance engineering, our approach attempted to exploit a microbial platform that has a natural tolerance to acid and numerous lignocellulose-derived inhibitors. *S. cerevisiae* BTCC3, an originally robust strain, was utilized as a fermentation host that was expected to be suitable for lactic acid production from lignocellulosic biomass. Pathway adjustment was conducted to reduce the metabolic flux to ethanol—a major product of fermentation by yeast—through the disruption of pyruvate decarboxylation gene, including *PDC1* and *PDC5*. However, although our strain could still maintain its rapid growth after several pathway adjustments, these genetic modifications also exerted several undesired effects. For instance, in all mutants, the accumulations of by-products, such as ethanol and glycerol, were higher than in wild-type strain, which could have been because of the response of the microbial host to cope with higher acid accumulations. In fact, the accumulations of ethanol and glycerol are known to induce the generation of NAD^+^, which has an essential role in countering the negative impact of various stressors in cells^24,42,43^. Also, our results revealed that inserting an additional copy of the *LDH* gene into the same locus does not necessarily improve lactic acid production. However, this could be the consequence of employing an identical promoter, i.e., *TDH3p*, in two different plasmids, namely pAUR101-TDH3pro-LcLLDH-dPDC1 and pAUR101-TDH3pro-LcLLDH-dPDC5. Promoter rivalry might have resulted in a conflict in the use of transcription factors during the expression of the two *LDH* genes. Moreover, we observed that the engineered strains grew slower when more genes were disrupted. For those reasons, keeping the genetic modifications at a modest level is much preferable rather than conducting immense gene modifications to the microbial host. In essence, with only a few pathway adjustments, we managed to enhance the accumulation of lactic acid from 0 to 43.23 g L^−1^ while still maintaining the natural feature of the strain to rapidly consume glucose.

As one of the most concerning bottlenecks in industrial lactic acid production, removing the neutralizing step during fermentation to enable the generation of free-form lactic acid that requires no subsequent acidification is important. In fact, according to the life-cycle assessment and techno-economic analysis of SCB valorization to lactic acid, the removal of neutralizing agents by employing an acid-tolerant host could reduce the environmental burden and total capital investment because the process would no longer require an acidification reactor unit set after the fermentation stage^44^. Also, the production of second-generation (2G) lactic acid from SCB, to date, still requisites the neutralizing step, either at the beginning or during the fermentation. Our results proved that BTCC3 strain could still grow without any neutralizing treatment both in low and high cell density cultivation. However, increasing the initial cell concentration could optimize the amount of glucose consumed for lactic acid accumulation. Further, LA2 strain could also facilitate a rapid neutralizing-agent-free lactic acid generation using undetoxified hydrolysate of SCB with a level of productivity that was competitive to other studies that still included additional treatment, such as neutralization, detoxification, and cell adaptation, in their experiment^38–41^. Based on our best knowledge, no previous reports discuss the SCB valorization to lactic acid that utilizes metabolically engineered strain as a host in this simplified setting. For upcoming experiments, constructing a switchable metabolic disruption strategy using an optogenetic tool^45^ or advanced CRISPR strategies may help knocking-out other gene candidates without severely reducing the rate of cell growth. Utilizing xylose as an additional carbon source by introducing a xylose-assimilating pathway is also essential as this sugar is the second most abundant monosaccharide present in various biomass. Also, BTCC3 strain can also be harnessed as a microbial factory to produce other important chemicals from various renewable sources.

In conclusion, sustainable lactic acid production is more efficient when several treatment stages can be omitted from the process. In this work, we demonstrated a rapid fermentation of lactic acid from hydrolysate of sugarcane bagasse without performing detoxification and neutralization steps. This process was catalyzed by an originally robust microorganism with only a few metabolic adjustments. This engineered host might be suitable for cost-effective and greener lactic acid production for industry.

## Materials and methods

### Strains and medium

*Saccharomyces cerevisiae* BTCC3 (haploid; registered as ID-Y003) was obtained from Indonesian Culture Collection (InaCC Cibinong Science Center BRIN; http://inacc.brin.go.id/), whereas *Saccharomyces cerevisiae* BY4741 (haploid) and Ethanol-red (diploid) were purchased from Funakoshi (Japan). All yeast strains were cultivated using yeast/nitrogen/base (YNB) (6 g L^−1^ YNB and 50 g L^−1^ glucose) as a minimum synthetic medium or yeast/peptone/dextrose (YPD) (20 g L^−1^ peptone, 10 g L^−1^ yeast extract, and 100 g L^−1^ glucose) as a rich medium. *Escherichia coli* JM109 (Takara Bio, Japan) was used for cloning and cultivated using Luria–Bertani (LB) medium (10 g L^−1^ tryptone, 5 g L^−1^ yeast extract, and 10 g L^−1^ sodium chloride).

### Robustness test

For single inhibitor tests at various concentrations, cells were cultivated using a minimum synthetic medium supplemented with a defined concentration of chemical inhibitor. Initially, a single colony was precultured using 12 mL YPD medium (30 °C, 150 rpm) overnight. Cells were adjusted to reach initial OD_600nm_ around 0.1 and cultivated using 1.2 mL YNB medium containing a single chemical inhibitor at a defined concentration in a 96-well plate (30 °C, 1500 rpm). Cell growth was measured periodically at a wavelength of 600 nm using a multilabel plate reader (2104 EnVision, PerkinElmer, USA). For combinatorial inhibitor tests, the cells obtained from preculture was adjusted to reach OD_600nm_ around 4.0 and cultivated using 12 mL YNB medium containing a combination of chemical inhibitors defined as ICC at 20% level (20%ICC), ICC at 100% level (ICC), and ICC at 100% level plus 0.6 mM vanillin and 0.4 mM syringaldehyde (ICC + VS). A medium supplemented with ICC at 100% level consisted of acetic acids, formic acid, furfural, 5-HMF, and levulinic acid at concentrations of 60 mM, 30 mM, 60 mM, 10 mM, and 5 mM, respectively, as described elsewhere^23^. Cell growth and glucose concentration were monitored periodically using a spectrophotometer at a wavelength of 600 nm (UVmini-1240, Shimadzu, Japan) and HPLC, respectively.

### Plasmid and strain construction

A codon-optimized L-*LDH* from *Lactobacillus casei* (GenBank accession number MF582630.1) was selected as the *LDH* gene source. All DNA fragments were assembled by following the NEBuilder HiFi DNA Assembly method (NEB, USA) and transformed into *E. coli* JM109. Plasmid-harboring transformants were cultivated overnight (35 °C, 200 rpm) using LB medium supplemented with 0.1 g L^−1^ of ampicillin as a selection marker. Plasmids were extracted using a LaboPass™ Plasmid Mini kit (Cosmo Genetech, Korea). Digested plasmids were transformed into a wild-type strain by following a LiAc/single-stranded carrier DNA/PEG method^46^. Each transformant was selected on a YPD agar containing selection marker(s).

### Fermentation

Cells were cultivated using a rich medium. Initially, a single colony was precultured using 12 mL YPD medium (30 °C, 150 rpm) for two days. Cells were adjusted to reach the desired initial OD_600nm_ and cultivated using 12 mL YPD medium (30 °C, 90 rpm). Two different conditions were studied: with calcium carbonate supplementation at the beginning of fermentation (semi-neutralized; SM) and without calcium carbonate supplementation (non-neutralized; NN). No pH adjustment was conducted during fermentation in both conditions. Moreover, recombinant cells were also cultivated using sterile-filtered sugarcane bagasse hydrolysate obtained from a pretreatment process following a method reported elsewhere^24^. After preculturing (30 °C, 150 rpm), cells were cultivated (30 °C, 90 rpm) in a 100-mL Erlenmeyer flask containing 12 mL of sterile hydrolysate without adding calcium carbonate.

### Analysis of fermentation products

Fermentation broths were periodically sample and centrifuged (10,000 rpm, 5 min). The supernatant was filtered through a 0.45-μM syringeless PTFE filter. Concentrations of glucose and other fermentation products were determined via HPLC (LC-20AB, Shimadzu, Japan) using a Coregel-87H3 column (7.8 mm, I.D. × 300 mm Transgenomic, USA) at 80 °C using 5 mM of sulfuric acid as eluent (flow rate at 0.6 mL min^−1^; 40 min). A refractive index detector was used.

### Statistical analysis

Values shown represent the mean of results from three biological replicates ± SD (standard deviation). Significant differences of lactic acid accumulated at the highest concentrations by the engineered strains were statistically compared using a two-tailed Student’s t-test at *P* ≤ 0.05.

## Supplementary Information

Supplementary Information.

## Data Availability

All data generated or analyzed during this study are included in this published article and its Supplementary Information file.
