# Supplementary material for: Harnessing originally robust yeast for rapid lactic acid bioproduction without detoxification and neutralization
Source: Sci Rep. 2022 Aug 11;12:13645. doi: 10.1038/s41598-022-17737-4 (PMC9372150; doi:10.1038/s41598-022-17737-4)
Supplement: Supplementary file 1 — Supplementary Information. [file 41598_2022_17737_MOESM1_ESM.pdf]

Supplementary Information

Harnessing originally robust yeast for rapid lactic acid bioproduction without detoxification and neutralization

Radityo Pangestu[1,2,+], Prihardi Kahar[1,+], Lutfi Nia Kholida[2], Urip Perwitasari[2], Ahmad Thontowi[2], Fahrurrozi[2], Puspita Lisdiyanti[2], Yopi[2,3], Chiaki Ogino[1,*], Bambang Prasetya[3], Akihiko Kondo[1,4]

[1]Graduate School of Engineering, Kobe University, 1-1 Rokkodaicho, Nada-ku, Kobe 657-8501, Japan
[2]National Research and Innovation Agency, Jl. Raya Bogor Km 46, Cibinong, 16911 Bogor, West Java, Indonesia
[3]National Standardization Agency of Indonesia (BSN), Gedung Badan Pengkajian dan Penerapan Teknologi (BPPT), Jl. M.H. Thamrin No. 8, Jakarta 10340, Indonesia
[4]Graduate School of Science, Technology, and Innovation (STIN), Kobe University, 1-1 Rokkodaicho, Nada-ku, Kobe 657-8501, Japan

[*]ochiaki@port.kobe-u.ac.jp
[+] these authors contributed equally to this work and share the main authorship

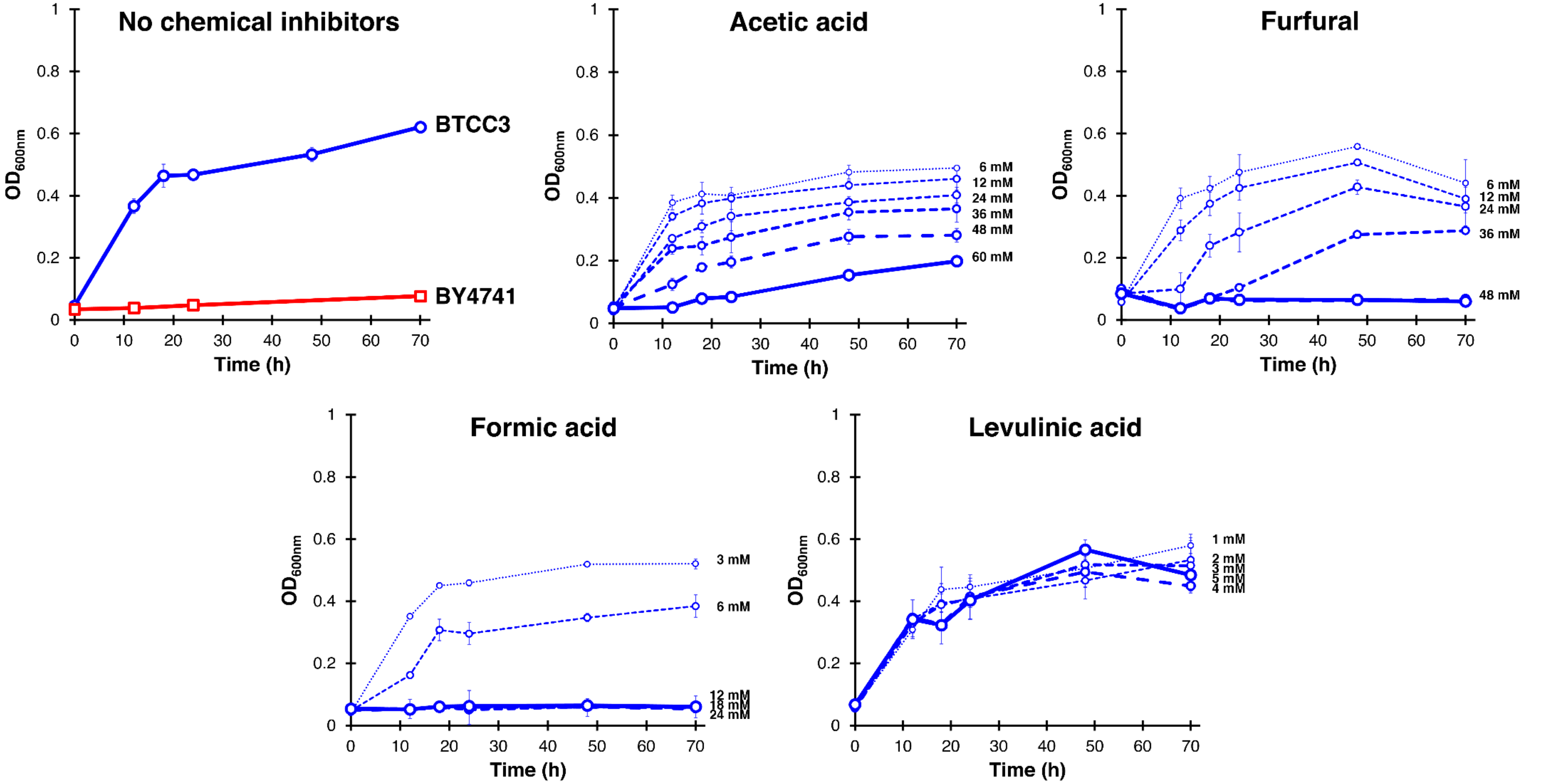

Supplementary figure 1: Comparison of the growth of Saccharomyces cerevisiae BTCC3 (blue circles; ○) and BY4741 (red boxes; □) strains in YNB medium without amino acids supplementation in the absence and presence of chemical inhibitors. Values represent the average measurement of three biological replicates. Error bars represent the standard deviation of measurements. Data for cultivation of BY4741 in the presence of chemical inhibitors are not shown due to insignificant growth within 70 h of cultivation.

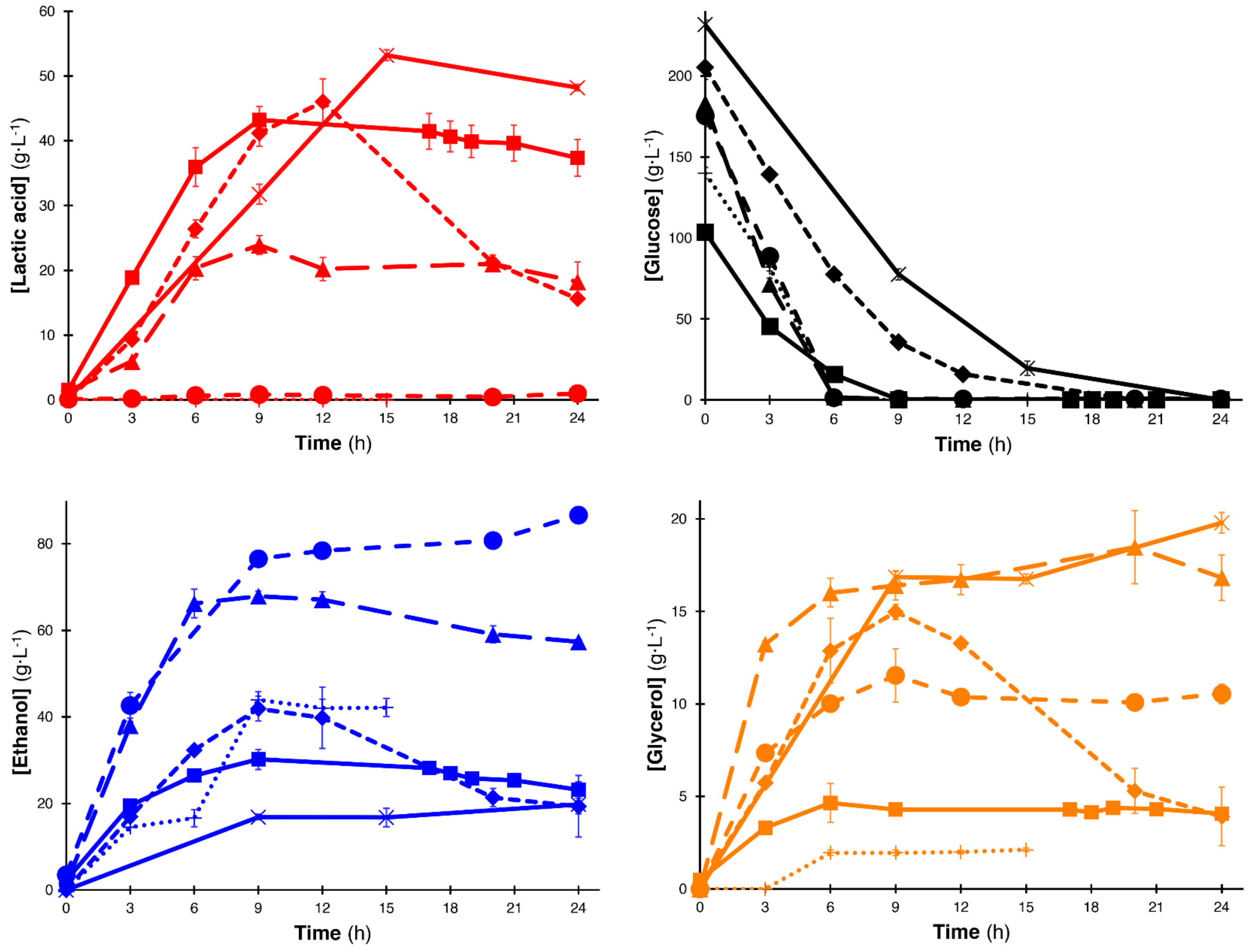

Supplementary figure 2: Comparison of major products produced by Saccharomyces cerevisiae BTCC3 wild-type (plus marks, +) and all its derived strains, including LX1 (closed triangles, ▲), LX5 (closed circles, ●), LA1 (closed diamonds, ◆), LA15 (closed X-marks, ✖) and LA2 (closed boxed, ■) strains, under semi-neutralized condition with the initial OD_{600nm} at 50. Values represent the average measurement of four biological replicates. Error bars represent the standard deviation of measurements.

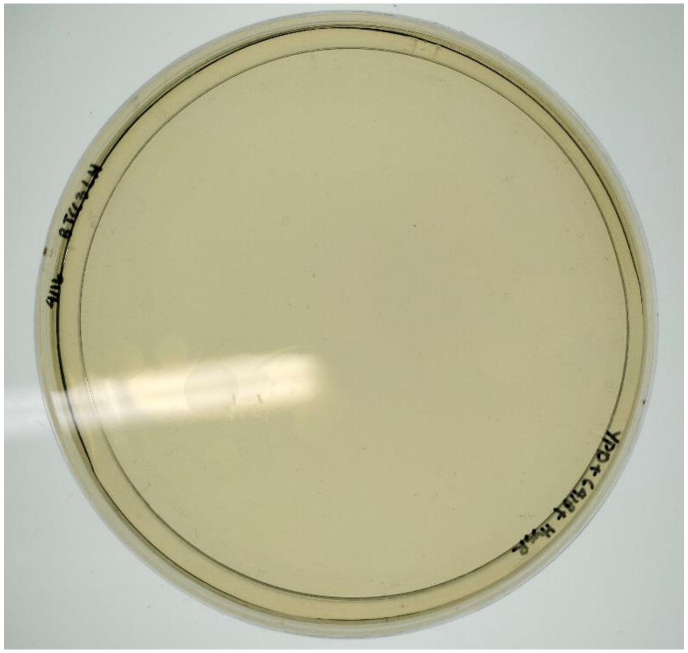

Supplementary figure 3: No transformants were obtained after simultaneously disrupting PDC1, PDC5, PDC6 and ADH1. The strain was engineered from BTCC3 wild-type strain. The LDH gene was inserted into CYB2 locus under the control of TDH3 promoter, whereas the four ethanol-related genes were disrupted using HI-CRISPR method proposed elsewhere[1].

Supplementary table 1: List of primers used in this study

Primer	Sequence	Description
P1-1.FOR<br>P1-1.REV	5’-CAGAGAATTTTTCAATCATTGGAGCAATCATTTTACA-3’<br>5’-GCATTTAAAAGATTATGTATGCTCTTCTGACTTTTCG-3’	Amplifying sequence of PDC1p-PDC1-PDC1t from genome for subsequent integration into marker genes-consisting plasmid
P1-2.FOR<br>P1-2.REV	5’-TCCTCTTTCATTGCAAGCCTTTTGGAGTTGAAGGTATGAGATGGGCT-3’<br>5’-GGATGCCCAGACTGCAGTTTGGGAGTCAAGTCAATCAACTTCTTAGTTTCAGC-3’	Amplifying partial RNA-coding sequence of PDC1 with the additional of overhang for integration into plasmid containing sequence of TDH3p-LcLLDH-TDH3t and generate pAUR101-TDH3pro-LcLLDH-dPDC1
P5-1.FOR<br>P5-1.REV	5’-CTGCAGGTCGACGGATCTGATTGTTGGATGCTATTCCAGAAGTCG-3’<br>5’-GTTATTAGGTGATAAGTTGGCAATAAGGCCAAGTGG-3’	Amplifying partial RNA-coding sequence of PDC5 with the additional of overhang for integration to generate pPC01-BTCC3PDC5KO
P5-2.FOR<br>P5-2.REV	5’-TCATGATAATAATGGTTTCTTAGACAACGCTAACGAATTGAACGCTG-3’<br>5’-CCCCGAAAAGTGCCACCTGACGGTGACGTAAACTGGGAATTGAGTCA-3’	Amplifying partial RNA-coding sequence of PDC5 with the additional of overhang for integration into plasmid containing sequence of TDH3p-LcLLDH-TDH3t and generate pAUR101-TDH3pro-LcLLDH-dPDC5
LLc-1.FOR<br>LLc-1.REV	5’-TAGAGATTAAATCGCTCATTGTCTTGTTTCAATGT-3’<br>5’-GTCAAATCAATCAAAATGGTTGCCT CCA-3’	Amplifying LcLLDH with the additional of overhang for integration to generate pAUR101-BTCC3PDC1-LcLLDH
LLc-2.FOR<br>LLc-2.REV	5’-GGAGGCAACCATTTTGTTTATTTATGTGTGTTTATTCGAAACTAAG-3’<br>5’-AACAAGACAATGAGTGAATTTACTTTAAATCTTGCATTTAAATAAATTTTCTTTTTATAGCT-3’	Linearizing plasmid containing sequences of TDH3p and TDH3t with the additional of overhang to integrate with LcLLDH
LLc-3.FOR<br>LLc-3.REV	5’- AATAAACAAAATGGTTGCCTCCATTACCGA-3’<br>5’-GTAAATTCACTCATTGTCTTGTTTCAATGTCGTTCTTGC-3’	Amplifying LcLLDH with the additional of overhang for integration into plasmid containing sequences of TDH3p and TDH3t

Note: The underlined bases are overlapping sequences to the genome.

REFERENCE

1. Bao, Z. et al. Homology-Integrated CRISPR–Cas (HI-CRISPR) System for One-Step Multigene Disruption in Saccharomyces cerevisiae. ACS Synth. Biol. 4, 585–594 (2015).
